# No evidence of inbreeding depression in sperm performance traits in wild song sparrows

**DOI:** 10.1002/ece3.3721

**Published:** 2018-01-12

**Authors:** Sylvain Losdat, Ryan R. Germain, Pirmin Nietlisbach, Peter Arcese, Jane M. Reid

**Affiliations:** ^1^ Institute of Biological and Environmental Sciences School of Biological Sciences University of Aberdeen Aberdeen Scotland; ^2^ Institute of Biology University of Neuchâtel Neuchâtel Switzerland; ^3^ Institute of Evolutionary Biology and Environmental Studies University of Zurich Zurich Switzerland; ^4^ Department of Zoology University of British Columbia Vancouver BC Canada; ^5^ Department of Forest and Conservation Sciences University of British Columbia Vancouver BC Canada

**Keywords:** genetic relatedness, inbreeding, paternity, reproductive strategies, sexual selection, sperm quality

## Abstract

Inbreeding is widely hypothesized to shape mating systems and population persistence, but such effects will depend on which traits show inbreeding depression. Population and evolutionary consequences could be substantial if inbreeding decreases sperm performance and hence decreases male fertilization success and female fertility. However, the magnitude of inbreeding depression in sperm performance traits has rarely been estimated in wild populations experiencing natural variation in inbreeding. Further, the hypothesis that inbreeding could increase within‐ejaculate variation in sperm traits and thereby further affect male fertilization success has not been explicitly tested. We used a wild pedigreed song sparrow (*Melospiza melodia*) population, where frequent extrapair copulations likely create strong postcopulatory competition for fertilization success, to quantify effects of male coefficient of inbreeding (*f*) on key sperm performance traits. We found no evidence of inbreeding depression in sperm motility, longevity, or velocity, and the within‐ejaculate variance in sperm velocity did not increase with male *f*. Contrary to inferences from highly inbred captive and experimental populations, our results imply that moderate inbreeding will not necessarily constrain sperm performance in wild populations. Consequently, the widely observed individual‐level and population‐level inbreeding depression in male and female fitness may not stem from reduced sperm performance in inbred males.

## 1 INTRODUCTION

Inbreeding and consequent inbreeding depression, defined as reduced mean fitness in offspring resulting from mating between relatives, is widely hypothesized to drive the evolution of mating systems and mate choice (Charlesworth, 2006; Szulkin, Stopher, Pemberton, & Reid, 2013; Tregenza & Wedell, 2000) and to increase population extinction risk (Crnokrak & Roff, 1999; Hedrick & Kalinowski, 2000; Kenney, Allendorf, McDougal, & Smith, 2014). However, the degree to which inbreeding could drive such population and evolutionary dynamics will depend on which life‐history traits and fitness components exhibit inbreeding depression.

Inbreeding could profoundly affect mating system and population dynamics if it caused severe inbreeding depression in primary sexual traits expressed by inbred individuals, including male gametic traits underlying sperm performance. Such inbreeding depression could reduce male and hence female fertilities and thereby reduce individual and population‐wide reproductive fitness (Pizzari & Parker, 2009; Snow & Spira, 1996). Furthermore, by reducing female fertility through sperm limitation, inbreeding depression in sperm traits could potentially drive evolution of female multiple mating (Bocedi & Reid, 2017; see also Birkhead & Pizzari, 2002; Forbes, 2014). As female multiple mating causes sperm competition (i.e., postcopulatory competition among different males’ sperm to fertilize ova, Parker, 1970), inbreeding depression in male sperm traits and fertilization success might then be exacerbated in an analogous way as precopulatory competition exacerbates inbreeding depression in male mating success (Joron & Brakefield, 2003; Meagher, Penn, & Potts, 2000). Inbreeding depression in male gametic traits might consequently cause inbreeding depression in individual fitness and hence cause indirect selection for females and males to avoid inbreeding through mate choice and/or dispersal.

In addition to reducing mean trait values, inbreeding might also be hypothesized to increase within‐ejaculate phenotypic variance in sperm traits. Inbreeding can increase among‐individual and among‐population variances in diverse phenotypic traits (Pray & Goodnight, 1997; Whitlock & Fowler, 1996), but might also be expected to increase within‐individual variance, for example, due to reduced developmental stability. In the context of sperm performance, inbred males might produce more variable sperm, for example, due to reduced control of the spermatogenesis process (reviewed in Losdat, Chang, & Reid, 2014). Increased within‐ejaculate variance in sperm phenotypic traits such as length, motility, and velocity has been hypothesized to reduce male fertilization success under sperm competition (Immler, Calhim, & Birkhead, 2008; Kupriyanova & Havenhand, 2002), and increased within‐individual variance in sperm longevity can cause negative transgenerational effects on offspring fitness (Immler, Hotzy, Alavioon, Petersson, & Arnqvist, 2014). Therefore, by affecting within‐individual variance in sperm traits, inbreeding might have greater effects on male fertilization success beyond those stemming solely from reduced mean trait value.

Inbreeding depression in mean male gametic traits has been demonstrated in domesticated animals and plants and in experimental populations bred under laboratory conditions. A recent review indicated a grand mean inbreeding load of approximately one haploid lethal equivalent across all studied sperm and pollen traits, although several studies showed little or no inbreeding depression (Losdat et al., 2014). However, most studies examined effects of severe inbreeding (i.e., one or multiple generations of selfing or sib–sib mating), which exceeds that commonly observed in nature in nonselfing species with obligate biparental reproduction. As inbreeding depression can be nonlinear and only expressed given severe rather than moderate inbreeding (e.g., Ala‐Honkola et al., 2013; Zajitschek, Lindholm, Evans, & Brooks, 2009), such studies might overestimate the magnitude of inbreeding depression expressed given degrees of inbreeding that commonly occur in wild nonselfing populations. Conversely, as captivity and benign environmental conditions often decrease inbreeding depression (Joron & Brakefield, 2003; Meagher et al., 2000), inbreeding depression in gametic traits expressed in wild populations might exceed that evident in domesticated and experimental populations. Consequently, to understand the implications of inbreeding for mating system evolution and population dynamics, the magnitude of inbreeding depression in male gametic traits arising in wild populations showing natural degrees of inbreeding should be quantified.

However, surprisingly few studies have examined effects of inbreeding on sperm traits in wild populations, and such studies have primarily focused on highly inbred populations. The percentage of morphologically abnormal sperm was greater in a highly inbred lion (*Panthera leo*) population with low population‐wide allozyme heterozygosity than in an adjacent larger, more heterozygous population (Wildt et al., 1987). Similarly, sperm abnormality decreased with microsatellite heterozygosity across and within European rabbit (*Oryctolagus cuniculus*) populations, particularly encompassing individuals from isolated island populations with very low heterozygosity (Gage et al., 2006). In contrast, multiple sperm traits did not vary with microsatellite heterozygosity across highly inbred cheetahs (*Acinonyx jubatus*), possibly because deleterious mutations had been fixed through severe historical population bottlenecks (Terrell et al., 2016). Studies that quantify effects of inbreeding on the mean and within‐ejaculate variance in sperm performance traits across individuals within populations experiencing more typical levels of inbreeding are therefore required.

Studies aiming to quantify inbreeding effects on male gametic traits must also consider other male attributes that may affect trait values and which are also of direct interest in the context of understanding mating system evolution. Specifically, a male's social status and consequent reproductive tactic might predict or affect its sperm performance (Pizzari & Parker, 2009). Under risk of sperm competition, males might trade‐off resources between traits that reduce sperm competition (e.g., increased mate guarding) versus traits that increase fertilization success (e.g., increased sperm quality, Kelly & Jennions, 2011; Schradin, Eder, & Müller, 2012). Such status‐dependent investment, where non‐mate‐guarding floater, satellite, or sneaker males exhibit better sperm performance than dominant mate‐guarding males, has been observed in captive and wild vertebrates (Fasel et al., 2016; Fitzpatrick, Desjardins, Milligan, Montgomerie, & Balshine, 2007; Froman, Pizzari, Feltmann, Castillo‐Juarez, & Birkhead, 2002; Neff, Fu, & Gross, 2003; Stockley, Searle, Macdonald, & Jones, 1994). Therefore, effects of individual social status should also be estimated when quantifying sperm performance in systems with state‐dependent plasticity in tactics.

We used wild pedigreed song sparrows (*Melospiza melodia*) to test the hypotheses that the mean and within‐ejaculate variance in sperm performance traits, respectively, decrease and increase with increasing male coefficient of inbreeding. Further, we tested whether the mean and variance in these traits differed between socially paired and socially unpaired males, which have different opportunities for mate guarding and might consequently experience different trade‐offs regarding sperm performance.

## 2 MATERIAL AND METHODS

### 2.1 Study system

A resident population of song sparrows inhabiting Mandarte Island (British Columbia, Canada) has been intensively studied since 1975; reproductive activity has been intensively monitored and all individuals hatched on Mandarte have been color‐ringed before fledging (Losdat, Arcese, & Reid, 2015; Smith, Keller, Marr, & Arcese, 2006). Mandarte's song sparrows are primarily socially monogamous; socially paired females and males defend territories and typically rear two or three broods of offspring during April–July every year, starting from age 1 year. However, there is substantial extrapair paternity; on average, 28% of offspring are sired by extrapair males, affecting 44% of broods (Sardell, Keller, Arcese, Bucher, & Reid, 2010). Males are consequently likely to experience substantial sperm competition resulting from female multiple mating, creating selection on the mean and variance in sperm traits (e.g., Immler et al., 2008; Kleven, Laskemoen, Fossoy, Robertson, & Lifjeld, 2008). Further, as the adult sex ratio is typically male‐biased (58% males during 2012–2014), some territorial adult males remain socially unpaired (Smith et al., 2006) and can hence only achieve reproductive success through extrapair paternity.

The population numbered 24–38 pairs during 2012–2014 and receives occasional immigrants (average 0.9 reproductive immigrants per year, Wolak & Reid, 2016). As Mandarte forms part of a large metapopulation, immigrants can be assumed to be unrelated to existing residents and to each other and consequently prevent the population‐wide degree of inbreeding from accumulating (Keller et al., 2001; Reid, Arcese, & Keller, 2006; Wolak & Reid, 2016). Although song sparrows do not substantively avoid inbreeding through nonrandom social pairing or extrapair reproduction, the frequency of close inbreeding (i.e., among first‐order relatives) is low given random mating (Keller, 1998; Reid et al., 2015). However, there is frequent inbreeding among second‐ and third‐order relatives, generating moderately inbred offspring (Reid et al., 2015; Wolak & Reid, 2016). Overall, the combination of a small resident core population with a low natural immigration rate generates substantial within‐population variation in inbreeding across the range that occurs widely in viscous populations (e.g., Hatchwell, 2010). The focal song sparrow system is therefore well suited to estimating inbreeding depression in key life‐history and physiological traits arising given natural patterns of inbreeding in vertebrate mating systems (Crnokrak & Roff, 1999; Reid, Arcese, Keller, & Losdat, 2014; Reid, Arcese, & Losdat, 2014; Reid et al., 2007).

### 2.2 Sperm sampling

To measure sperm traits, we mist‐netted male song sparrows on their territories during April 23rd to May 23rd in 2012, 2013, and 2014 (i.e., early in each breeding season). Each male was sperm sampled and released back in its territory within ten minutes. Song sparrows’ laying dates are highly asynchronous, such that at any point throughout the catching period, some females were likely to be fertile, thereby continuously providing opportunities for males to obtain paternities.

Sperm samples were collected by gently massaging males’ cloacal protuberance (Wolfson, 1952). Collected sperm (ca. 1 μl) were mixed immediately with prewarmed (40°C) Dulbecco's modified Eagle's medium (4,500 mg glucose/L, 110 mg sodium pyruvate/L, 4 mM L‐glutamine, Sigma‐Aldrich, UK). A 9‐μl aliquot of sperm/Dulbecco solution was then deposited on a slide and immediately transferred to a dark‐field phase‐contrast microscope, where sperm motion was video‐recorded for 5 min during which the sample was maintained at 40°C, following standard protocols (e.g., Lifjeld et al., 2013). Temporal dynamics of sperm motion were analyzed after 0, 1, 2, 3, 4, and 5 min of video recording from a video segment of 3s at each time point, using a computer‐assisted sperm analysis plug‐in implemented in ImageJ software (Wilson‐Leedy & Ingermann, 2007). Sperm cells slower than 5 μm/s were considered immotile or moved by drift. Measuring sperm performance in vitro right after ejaculation has proved biologically relevant in internally fertilizing species. For example, sperm velocity measured in vitro predicts male fertilization success in several species (see table 2 in Simmons & Fitzpatrick, 2012).

We quantified three standard sperm traits: sperm motility defined as the relative numbers of motile vs. not motile sperm at a focal time point, sperm longevity measured as the rate of decrease in sperm motility across the 0–5 min of video recording, and the straight‐line velocity of motile sperm at time 0. These traits predict male fertilization success in diverse species (Boschetto, Gasparini, & Pilastro, 2011; Denk, Holzmann, Peters, Vermeirssen, & Kempenaers, 2005; Gage et al., 2004; Malo et al., 2005; reviewed in Fitzpatrick & Lüpold, 2014; Simmons & Fitzpatrick, 2012;) and are consequently likely to be under directional selection and hence to show inbreeding depression (e.g., Crnokrak & Roff, 1999; Lynch & Walsh, 1998). We also quantified the within‐ejaculate variance in sperm velocity at time 0 as the coefficient of variation in the velocities of motile sperm (CV_velocity_ = *SD*_velocity_/MEAN_velocity_, Immler et al., 2008; Kleven et al., 2008). We did not observe the kind of sperm abnormalities commonly reported in mammals (double‐headed sperm or biflagellated sperm, e.g., Gage et al., 2006) and in captive songbirds (atypical helical head shape, tail deformities, two‐tailed sperm, e.g., Opatová et al., 2016). We focused on metrics of sperm swimming ability because those traits predict fertilization success in several internal fertilizing species (e.g., table 2 in Simmons & Fitzpatrick, 2012), hence allowing relatively straightforward evolutionary inference.

### 2.3 Coefficient of inbreeding and pairing status

During 1993–2014, 99.7% of all song sparrows hatched on Mandarte, and all immigrants, were blood sampled and genotyped at 160 microsatellite loci (Nietlisbach et al., 2015). All Mandarte‐hatched individuals were assigned to their true genetic parents with >99% individual‐level statistical confidence, allowing reconstruction of a complete genetic pedigree (e.g., Sardell et al., 2010). This genetic pedigree was combined with parentage inferred from comprehensive observations of social pairings spanning 1975–1992 to compile a full pedigree covering 1975–2014 (Losdat et al., 2015; Reid, Arcese, Keller, et al., 2014; Reid, Arcese, & Losdat, 2014; Sardell et al., 2010). We applied standard algorithms to the full pedigree to calculate each male's coefficient of inbreeding *f*, which is defined as the probability that two homologous alleles will be identical by descent relative to the pedigree baseline, and therefore measures relative expected genome‐wide homozygosity. For example, *f *=* *0.0625 and *f *=* *0.125 correspond to males whose parents were third‐order and second‐order relatives, respectively (e.g., first cousins and half‐sibs). The males whose sperm was sampled during 2012–2014 had hatched during 2007–2013. Consequently, all nonimmigrant ancestors back to great‐grandparents, 98% of great‐great‐grandparents, and 88% of great‐great‐great‐grandparents were genetically verified. Individual *f* values were therefore estimated with negligible error (Reid et al., 2015). Further, the mean maximum depth of the full 1975–2014 pedigree across the sampled males was 23 generations (range: 20‐25). Offspring of immigrant–native pairings are defined as outbred relative to the Mandarte pedigree baseline (*f *=* *0, Reid et al., 2006). However, immigrants’ own *f* values are undefined relative to this baseline. Two immigrant males whose sperm was sampled were therefore excluded from the analyses, but their trait values are included in figures for visual comparison with Mandarte‐hatched males.

Males were classified as “socially paired” if they were paired with a female (i.e., displaying mate‐guarding and chick‐feeding behavior) at the time of sperm sampling or as “unpaired” if not (Losdat et al., 2015; Smith et al., 2006).

### 2.4 Statistical analyses

To test whether sperm performance traits varied with male *f* or pairing status, we fitted four separate generalized linear mixed models. Dependent variables were sperm motility at time 0 (one value per ejaculate), sperm motility at all time points (six values per ejaculate, allowing estimation of sperm longevity), sperm velocity (one value per motile sperm within each ejaculate), and CV_velocity_ (one value per ejaculate). All models included random male identity effects to account for nonindependence among samples of males captured more than once across years. The sperm velocity model additionally included random ejaculate effects (nested within male identity) to account for nonindependence among individual sperm within an ejaculate.

All models included fixed regressions on male *f* and fixed effects of male pairing status (two‐level factor, socially paired or unpaired) and year (three‐level factor). Sperm performance traits might also vary with individual age (Pizzari, Dean, Pacey, Moore, & Bonsall, 2008). Indeed, older males have been shown to produce lower‐quality sperm in laboratory and captive vertebrates (Gasparini, Marino, Boschetto, & Pilastro, 2010; Preston, Jalme, Hingrat, Lacroix, & Sorci, 2011; Wolf et al., 2000), but such age‐specific variation has rarely been shown in wild populations (but see Møller et al., 2009). We did not have sufficient longitudinal data to rigorously quantify within‐male age effects. However, to account for any such effects, all models additionally included a fixed regression on male age, which was known because all (nonimmigrant) males had been ringed as chicks. All models also included *f*‐by‐pairing status, *f*‐by‐age, and *f‐*by‐year interactions to test whether the magnitude of inbreeding depression depended on these parameters. Models also included fixed regressions on sampling date within year (Julian date) to control for any associated variation. We also initially tested for effects of the minutes elapsed between sperm collection and the time 0 start of video recording but as no such effects were detected, this variable was excluded from the final models. The model of sperm longevity included a fixed regression on time since start of video recording and interactions of time by *f*, time by pairing status, time by age, and time by motility at time 0. These interactions test for effects of male *f*, pairing status, age, and initial sperm motility on the rate of decrease in motility.

Sperm motility and longevity were modeled as binomial traits with the numbers of motile sperm and total sperm assayed as numerator and denominator, respectively. Because these two models required accounting for overdispersion, they were fitted in a Bayesian MCMC framework, which allows estimating residual variance. Models of sperm velocity and CV_velocity_ were fitted in a frequentist framework using restricted maximum‐likelihood estimation, assuming Gaussian error structures. Because log(fitness) is expected to decrease linearly with individual *f* (given multiplicative allelic effects, Keller & Waller, 2002), we modeled the logarithm of sperm velocity, meaning that the estimated slope directly equates to the (haploid) inbreeding load (Keller & Waller, 2002). Conclusions were similar when raw velocity values were modeled (data not shown). Finally, we fitted an additional linear model to estimate the slope of the regression of log(motility) on *f* and thereby directly estimate “sperm lethal equivalents*.”*

The number of individual sperm whose velocities were assayed varied among males, partly because sperm motility varied substantially among males (Figure S1). Therefore, to ensure that CV_velocity_ was adequately estimated, we excluded 10 males from whom <10 motile sperm were tracked, a threshold at which males’ CV_velocity_ values approximately reached an asymptote (Figure S2). No such cutoff was applied to the other sperm variables, either because all individual sperm values were modeled (sperm velocity), or because the number of sperm tracked was explicitly modeled as the binomial denominator (sperm motility and longevity). Mean sperm velocity was not correlated with the number of sperm whose velocities were estimated (r < .01, *p* = .24). Repeat estimation of sperm motility and velocity from each video at time 0 yielded very high measurement repeatability (motility: 0.96, velocity: 0.88).

Model reduction was limited to removing nonsignificant interactions (Whittingham, Stephens, Bradbury, & Freckleton, 2006); parameters were hence estimated in final models containing all fixed effects and interactions considered significant (i.e., *p* < .05 in frequentist models or 95% confidence intervals excluding 0 in Bayesian models). Additional modeling showed that quadratic effects of *f* and age were not significant and their inclusion did not change the model outputs. Analyses were run in R 3.3.2 (R Core Team, 2016) using “MCMCglmm” (Hadfield, 2010) and “lme4” (Bates, , Maechler, Bolker & Walker, 2014) packages. MCMCglmm models were run with default diffuse normal priors on fixed effects and parameter‐expanded priors on variance components with 1,005,000 iterations, burn‐in 5000, and thinning interval 1000. Posterior distributions were similar when models were rerun using inverse Wishart priors on variance components. Raw means are presented ±1 *SD*.

## 3 RESULTS

### 3.1 Data structure

**Figure 1:**
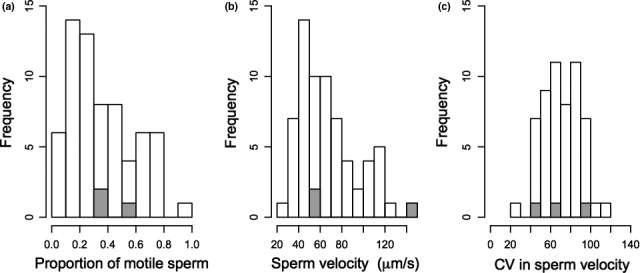
Distributions of (a) sperm motility (i.e., the proportion of sperm that were motile), (b) sperm velocity, and (c) coefficient of variation (CV) in sperm velocity at time 0. White and gray bars indicate values for Mandarte‐hatched and immigrant males, respectively. Mean ± *SD* trait values for Mandarte‐hatched males are (a) 0.36 ± 0.22, (b) 67.0 ± 27.6, and (c) 71 ± 18

Sperm performance was measured in 54 Mandarte‐hatched males totaling 66 observations (30, 20, and 16 in 2012, 2013, and 2014, respectively), and representing 78% of all adult males present on Mandarte during 2012‐2014. Seven of these males (nine observations) were offspring of immigrant–native pairings and therefore defined as outbred. Two immigrant males (three observations) were additionally sampled, and their sperm trait values generally fell within the ranges observed for native males (Figure 1). Of the 54 sampled males, eight were sampled in two different years and two were sampled in all 3 years.

Across the 66 samples, the mean total number of sperm assayed for motility at time 0 was 450 ± 501 (median: 249, Figure S1) and the mean number of motile sperm assayed for velocity was 238 ± 365 (median 79, Figure S1). Sperm motility, longevity, velocity, and CV_velocity_ all varied substantially among samples (Figure 1 and Figure S3).

Mean coefficient of inbreeding *f* across all 54 native males was 0.072 ± 0.046, which equates to offspring of matings between inbred third‐order relatives such as first cousins. However, individual *f* values ranged from 0.000 to 0.171 (Figure S3), which ranges from outbreeding to mating between inbred second‐order relatives (i.e., half‐siblings), hence spanning the range that commonly arises in wild vertebrates. At the time of sampling, 44 males were socially paired while 22 were unpaired. Of the ten males sampled in multiple years, only three changed status between samples. Mean male age was 1.8 ± 1.0 years (range 1‐5, Figure S3). Socially paired and unpaired males did not differ significantly with respect to *f* (Wilcoxon test: W = 409, *p* = .31), but socially paired males were typically older than unpaired males (mean ages: 2.0 and 1.3, medians: 2 and 1 years old, respectively, W = 706, *p* = .001).

### 3.2 Sperm performance, inbreeding, and pairing status

**Figure 2:**
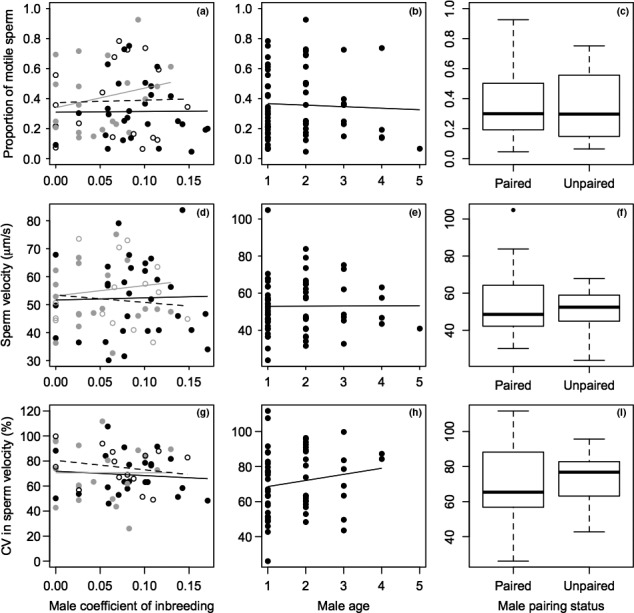
Variation in (a–c) sperm motility, (d–f) sperm velocity, and (g–i) coefficient of variation in sperm velocity (CV_velocity_) in relation to male (a, d, g) coefficient of inbreeding *f,* (b, e, h) age, and (c, f, i) pairing status. Each point represents one observation of sperm performance (total 66 from 54 different males for motility and velocity and total 56 from 48 different males for CV_velocity_). In (a, d, g), data from 2012, 2013, and 2014 are shown by black, gray, and white circles, respectively, and lines represent regression lines fitted across all observations in 2012 (black), 2013 (gray), and 2014 (dashed), for illustration. In (b, e, f), lines are regression lines fitted across all observations. In (c, f, i), boxplots show medians, first and third quartiles and whiskers correspond to 1.5 times the interquartile range

Sperm motility measured at time 0 did not vary significantly with male *f*, pairing status, or age (Table 1, Figure 2) or with any of the interactions (Table S4). However, motility tended to be lower in 2012 than in 2013 and 2014, and increased with Julian date (Table 1). Sperm longevity (i.e., the decrease in motility over the series of time points) also did not vary with male *f* as shown by the nonsignificant *f*‐by‐time interaction (Table 1, Figure S5). The main effects of year and Julian date on longevity were significant and, as expected, there was a significant effect of time since sampling (Table 1, Figure S5). Further, sperm velocity and CV_velocity_ measured at time 0 did not vary significantly with male *f*, pairing status, or age (Table 2, Figure 2), or with any of the interactions (Table S6). The estimated inbreeding load in sperm motility (i.e., “sperm lethal equivalents”) was 0.35 (95% CI −1.40–2.10), and the estimated inbreeding load in sperm velocity was 0.05 (95% CI −0.65–0.75, Table 2). Across the 10 males sampled more than once, we estimated repeatability of motility and velocity using Gaussian mixed models with individual male identity fitted as random effect and no fixed effect. Repeatability, the ratio of the random effect variance for male identity divided by the sum of the variance for male identity and residual variance (Nakagawa & Schielzeth 2010), was low (motility: r=.18, velocity: r=.09), partly reflecting the detected effects of year and Julian date.

**Table 1:** Bayesian generalized linear mixed models testing for effects of male coefficient of inbreeding (*f*), pairing status, year, age, and Julian date on (A) sperm motility at time 0 and (B) sperm longevity (i.e., the decrease in sperm motility with time). In (B), the model also includes effects of time and sperm motility at time 0 and interactions of time by *f*, time by mating status, time by age, and time by motility at time 0. Values are posterior means, 95% highest posterior density intervals (HPD), and *p*‐values based on posterior distributions (*p*MCMC). For (A), estimates for nonsignificant interactions (*f* by age, *f* by pairing status, and *f* by age) are shown in Table S4

Effect	(A) Sperm motility		(B) Sperm longevity	
	Posterior mean (95% HPD)	*p*MCMC	Posterior mean (95% HPD)	*p*MCMC
(Intercept)	−10.4 (−14.9 to −5.7)	‐	−8.2 (−11.3–5.3)	‐
*f*	1.1 (−6.0–7.5)	0.73	4.2 (−2.8–10.9)	0.56
Pairing status^a^	0.3 (−0.2–0.8)	0.33	−0.2 (−0.5–0.2)	0.24
Year				
(2013)^b^	0.7 (0.1–1.2)	0.02	0.3 (0.02–0.63)	0.05
(2014)^b^	0.6 (−0.1–1.3)	0.08	0.7 (0.1–1.2)	0.01
Age	−0.02 (−0.3–0.3)	0.90	−0.03 (−0.3–0.2)	0.86
Julian date	0.07 (0.04–0.11)	<0.002	0.05 (0.03 to −0.08)	0.001
Time	‐	‐	−0.003 (−0.004 to −0.002)	0.001
Motility time 0	‐	‐	0.04 (−0.49–0.45)	0.96
Time × Motility time 0	‐	‐	<0.002 (−0.002–0.002)	0.68
Time × *f*	‐	‐	0.02 (−8.1–0.01)	0.78
Time × Mating status^a^	‐	‐	<0.001 (−0.001–0.01)	0.92
Time × Age	‐	‐	<0.001 (−0.007–0.0005)	0.94

a Unpaired males relative to socially paired males.

b Relative to 2012.

**Table 2:** Linear mixed models testing for effects of male coefficient of inbreeding (*f*), pairing status, age, year, and Julian date on log‐transformed sperm velocity and on the coefficient of variation in sperm velocity

Effect	Sperm velocity			Coefficient of variation in sperm velocity		
	Estimate (95% CI)	*F*_df_	*p*	Estimate (95% CI)	*F*_df_	*p*
(Intercept)	1.57 (1.08–2.07)	‐	‐	5.1 (−81.5–91.8)	‐	‐
*f*	0.05 (−0.67–0.75)	0.02_1,55_	.89	−30.0 (−153.0–94.4)	0.20 _1,39_	.66
Pairing status^a^	−0.009 (−0.07–0.05)	0.08_1,56_	.78	5.83 (−5.37–17.0)	0.89 _1,49_	.35
Age	−0.00003 (−0.03–0.03)	0.001_1,64_	.99	4.24 (−2.3–10.7)	1.37 _1,48_	.25
Year						
(2013)^b^	(−0.05–0.08)	0.14_2,50_	.87	−0.56 (−11.8–10.7)	0.22 _2,37_	.80
(2014)^b^	−0.005 (−0.07–0.06)			3.66 (−8.9–16.2)		
Julian date	0.0004 (−0.003–0.004)	0.04_1,53_	.85	0.46 (−0.2–1.1)	1.50_1,49_	.23

Estimates are shown with 95% confidence intervals (CI) and associated *F*‐ and *p*‐values. Degrees of freedom were calculated using the Kenward–Roger approximation. Estimates for nonsignificant interactions and random effect(s) are shown in Table S6.

a Unpaired males relative to socially paired males.

b Relative to 2012.

## 4 DISCUSSION

The hypothesis that inbreeding depression could drive ongoing evolution of mating systems and associated traits requires the occurrence of inbreeding depression in key reproductive traits across the range of coefficients of inbreeding (*f*) generated by natural mating systems. However, we found no evidence of inbreeding depression in sperm performance across the natural range of *f* in wild song sparrows, measured as reduced sperm motility, longevity, or velocity. Further, we found no evidence that the within‐ejaculate coefficient of variance in sperm velocity increased significantly with increasing *f*, and hence that inbreeding increased the within‐individual variance in gametic trait expression.

While testing key biological hypotheses requires quantification of inbreeding depression across naturally occurring ranges of *f*, such ranges often mean that power to detect inbreeding effects in wild populations is low, for example, because of little variance in *f* (Keller & Waller, 2002). However, our study encompassed moderately inbred individuals alongside outbred offspring of immigrants. Consequently, the 95% confidence intervals around the estimated regression slopes of sperm motility and velocity on *f* were relatively narrow. The lower 95% confidence limits show that the minimal slope values we could have detected were, respectively, ‐0.65 and ‐1.40 for velocity and motility, equating to mild inbreeding depression (i.e., reductions of ~2 μm/s in sperm velocity or ~3% in sperm motility between males of *f *=* *0 and *f *=* *0.072). We could consequently have detected the inbreeding loads that were detected in sperm velocity in zebra finches (B = −1.34, Opatová et al., 2016) and in sperm motility across previous studies (B = −1.37, Losdat et al., 2014) as these values are close to or outside our lower 95% confidence limits. The lack of evidence of statistically significant inbreeding depression in our study hence does not simply reflect insufficient statistical power to detect biologically reasonable effects. Indeed, inbreeding depression in other key physiological traits and fitness components (e.g., immune responses, song repertoire size) has been detected in the focal song sparrow population given similar sample size with similar variance in *f (*Losdat, Arcese, Sampson, Villar, & Reid, 2016; Reid et al., 2005, 2007).

Multiple sperm performance traits are likely to be influenced by a male's diploid genotype more than the sperm's own haploid genotype (Losdat et al., 2014; Pizzari & Parker, 2009), and likely to be correlated with male reproductive success and hence under directional selection (Birkhead, Martinez, Burke, & Froman, 1999; Hunter & Birkhead, 2002; Lynch & Walsh, 1998). Consequently, the lack of inbreeding depression apparent in song sparrows is perhaps surprising and contrasts with general evidence of inbreeding depression in male sperm trait values across domesticated and experimental animal and plant species (Losdat et al., 2014). On Mandarte, although the population size is small, the immigration rate is sufficient to maintain substantial genetic variation and to prevent inbreeding from reaching severe levels (Keller et al., 2001). Further, observed sperm trait values fall within the range observed in other passerine species that also have moderate rates of extrapair paternity. For example, the mean song sparrow sperm velocity of 67.0 μm/s compares to 60 μm/s in wild barn swallows (*Hirundo rustica*, Møller et al., 2009) or 68 μm/s in wild house sparrows (Losdat, unpublished). There is consequently no evidence that Mandarte's song sparrows have remarkably or uniformly low sperm performance trait values due to high population‐wide inbreeding, as observed in wild lion and rabbit populations (Gage et al., 2006; Wildt et al., 1987) and inferred in cheetahs (Terrell et al., 2016). Population‐wide inbreeding is therefore unlikely to explain why inbreeding effects were not observed across contemporary variation in *f* among male song sparrows. Indeed, neither the seven outbred offspring of immigrant–native pairings nor the two immigrant males themselves showed systematically higher sperm trait values than offspring of native–native pairings (Figure 1).

In the wider context, recent studies on captive and experimental populations that estimated inbreeding depression in sperm traits given moderate inbreeding (i.e., that could commonly arise in wild vertebrate populations) showed inconsistent results. Mean sperm velocity decreased by ‐3.3 μm/s in inbred red bulls (*Bos taurus*,* f *=* *0.13, Dorado et al., 2015), by ‐12.7 μm/s in experimentally inbred wild‐caught zebra finches (*f *=* *0.25, Opatová et al., 2016), but there was no effect of inbreeding on sperm velocity, motility, or longevity in inbred captive lake trout (*Salvelinus namaycush*,* f *=* *0.125–0.25, Johnson, Butts, Smith, Wilson, & Pitcher, 2015). Together with our results, this evidence implies that moderate inbreeding by parents does not always result in sons with low sperm performance, at least considering some key sperm traits that can affect male reproductive success (reviewed in Pizzari & Parker, 2009; Fitzpatrick & Lüpold, 2014). However, there may still be a nonlinear relationship between sperm trait values and *f*, where inbreeding expression could be manifested and/or detectable only at very high *f* values that exceed those observed in song sparrows. This scenario was observed in guppies (*Poecilia reticulata*) and in *Drosophila melanogaster* where measures of sperm competitiveness showed inbreeding depression at *f *>* *0.50 but not at *f *=* *0.25 (Ala‐Honkola et al., 2013; Zajitschek et al., 2009). Therefore, unlike other physiological fitness‐related traits, inbreeding depression in sperm performance might only be manifested following relatively severe inbreeding.

The apparent absence of inbreeding depression in sperm performance across degrees of inbreeding that might commonly occur in wild populations of nonselfing organisms has implications for the mechanisms causing variation in reproductive success and associated mating system evolution. Strong inbreeding depression has been observed in major components of male fitness, including extrapair reproductive success in song sparrows (Losdat et al., 2015; Reid, Arcese, Keller, et al., 2014; Reid, Arcese, & Losdat, 2014) and annual breeding success in polygynous red deer *Cervus elaphus* (Huisman, Kruuk, Ellis, Clutton‐Brock, & Pemberton, 2016). The lack of inbreeding depression in sperm traits that presumably affect male fertilization success suggests that inbreeding depression in male reproductive success in song sparrows might primarily stem from inbreeding depression in traits that affect precopulatory processes and mating success (e.g., mating behavior and secondary sexual signals, Reid et al., 2005) rather than postcopulatory processes. It also implies that inbreeding depression might not substantially affect population dynamics through reductions in sperm performance, or substantially affect the fertility of females that mate with inbred males. However, it is possible that there is inbreeding depression in other key sperm traits that are difficult to measure in the wild, including sperm quantity, differential sperm allocation among mates, ejaculate fluid composition, and/or the sperm–ovarian fluid interactions; such effects remain largely untested. Further, although within‐species relationships between sperm morphology and fertilization success remain unclear (Simmons & Fitzpatrick, 2012), it would also be interesting to quantify inbreeding depression on sperm morphology in future studies.

Socially paired and unpaired male song sparrows did not differ in mean sperm trait values or within‐ejaculate variance in sperm velocity. There was therefore no evidence of differential investment in sperm performance across males with different social status and hence different reproductive tactics. This may reflect strong selection acting on sperm traits across all males in a polyandrous system with consequent widespread sperm competition (Fitzpatrick & Lüpold, 2014; Pizzari & Parker, 2009). Interestingly, unpaired male song sparrows generally sire fewer extrapair offspring than expected given their frequency in the population (Sardell et al., 2010). The absence of a difference in sperm traits between socially paired and unpaired males hence suggests that the lower extrapair reproductive success of unpaired individuals stems from reduced mating success and/or cryptic female choice rather than reduced sperm performance.

## CONFLICT OF INTEREST

None declared.

## AUTHORS’ CONTRIBUTIONS

SL designed the study, collected field data, carried out statistical analyses, and drafted the manuscript; JMR contributed to the study design, statistical analyses, and manuscript writing; RRG managed the long‐term database and contributed to fieldwork; PN performed the microsatellite genetic analyses and contributed to fieldwork; and PA coordinated the study and ran the long‐term study system. All authors gave final approval for publication.

## DATA ACCESSIBILITY

The datasets supporting this article can be accessed in the following repository: https://doi.org/10.5061/dryad.fg189
