# Supplementary material for: No evidence of inbreeding depression in sperm performance traits in wild song sparrows
Source: Ecol Evol. 2018 Jan 12;8(3):1842–52. doi: 10.1002/ece3.3721 (PMC5792576; doi:10.1002/ece3.3721)
Supplement: Supplementary file 1 [file ECE3-8-1842-s001.docx]

**Supporting Information**

**“***No evidence of inbreeding depression in sperm performance traits in wild song sparrows***”**

**Figure S1.** Frequency distribution of (a and b) total number of sperm assayed per ejaculate (data used in the sperm motility and longevity analyses) and (c and d) total number of motile sperm per ejaculate (data used in the sperm velocity analyses). For ease of visualization, a and c show all individual ejaculates whereas b and d show only ejaculates that had a small number of sperm. On (d) the vertical black line shows the threshold at which ejaculates with a smaller number of motile sperm were excluded from the analysis of the coefficient of variation in sperm velocity (n=10).

**Figure S2**. Coefficient of variation in sperm velocity in relation to the number of motile sperm used to calculate it. We used sperm velocities from 26 males from whom >100 motile sperm were tracked. For those males, we randomly chose 5, 10, 15, 20, 30, 50 or 100 of each male’s observed velocity values and further calculated CV_velocity_ based on each of these samples (seven estimations of CV_velocity_ per male). We chose to exclude males that had <10 motile sperm from the analysis on the coefficient of variation in sperm velocity as providing the best balance between adequately estimating CV_velocity_ while maximising available sample sizes.

**Figure S3.** Distributions of (a) sperm longevity (i.e. the change in the proportion of sperm that were motile per minute), (b) male coefficient of inbreeding *f* and (c) male age. In (a), white and grey bars indicate native and immigrant males, respectively. In (b) and (c), white and grey bars represent socially-paired and unpaired males, respectively. In (a), each longevity value corresponds to the slope of a linear regression of sperm motility across the video sampling times (0-5 minutes). The mean±1SD for Mandarte-hatched males is -3.3±2.8%.min^-1^.

**Table S4.** Bayesian generalized linear mixed model testing for effects of male coefficient of inbreeding (*f*), pairing status, age, year and Julian date, and *f* by pairing status, *f* by age and *f* by year interactions on sperm motility at time 0. Values for interactions are posterior means, 95% highest posterior density intervals (HPD) and *p*-values based on posterior distributions (*p*MCMC). The random effect value is the posterior mean and its HPD. The table shows values for the interactions only since main effects are shown in main text (Table 1).

| Effect | Posterior mean (95% HPD) | *p*MCMC |
| --- | --- | --- |
| *f* × Pairing status^a^ | 8.1 (-6.5-19.8) | 0.20 |
| *f* × Age | -2.5 (-8.7-4.3) | 0.48 |
| *f* × Year (2013)^b^ | -5.9 (-16.3-7.5) | 0.28 |
| (2014)^b^ | -2.2 (-15.7-11.0) | 0.77 |
|  |  |  |
| *Random effects*  Male |  |  |
| Male identity | 0.94 (0.14-1.55) |  |

^a^unpaired males relative to socially-paired males; ^b^relative to 2012

**Figure S5.** Decrease in sperm motility with time (i.e. sperm longevity) in relation to male coefficient of inbreeding *f*. For ease of graphical visualisation, males are pooled into three categories; 0.00 < *f ≤* 0.04 (grey, n=17 observations), 0.04 < *f ≤* 0.08 (black, n=18 observations) and *f* > 0.08 (white, n=31 observations). Each point represents mean sperm motility for a given time and *f* category. Whiskers show standard errors. The total numbers of sperm sampled at 0, 1, 2, 3, 4 and 5 minutes after start of video recording were 450, 428, 393, 370, 357, 351, respectively.

**Table S6.** Linear mixed models testing for effects of male coefficient of inbreeding (*f*), pairing status, age, year, Julian date and *f* by pairing status, *f* by age and *f* by year interactions on log-transformed sperm velocity and on the coefficient of variation in sperm velocity. Estimates for interactions are shown with their 95% confidence intervals (CI) and associated *F*- and *P*-values. Degrees of freedom were calculated using the Kenward-Roger approximation. Estimates for random effects are shown with their 95% CI. The table shows values for the interactions only since main effects are shown in main text (Table 3).

|  | **Sperm velocity** | | | **Coefficient of variation in sperm velocity** | | |
| --- | --- | --- | --- | --- | --- | --- |
| Effect | Estimate (95% CI) | *F*_df_ | *P* | Estimate (95% CI) | *F*_df_ | *P* |
| *f* × Pairing status^a^ | -0.26 (-1.79-1.41) | 0.09_1, 58_ | 0.77 | 143.3 (-205.7-424.8) | 0.75 _1, 45_ | 0.39 |
| *f* × Age | 0.07 (-0.72-0.94) | 0.02 _1, 67_ | 0.88 | -101.8 (-125.7-92.2) | 0.71 _1, 42_ | 0.40 |
| *f* × Year | - | 0.70 _2, 49_ | 0.50 | - | 0.22 _2, 36_ | 0.80 |
|  |  |  |  |  |  |  |
| *Random effects* |  |  |  |  |  |  |
| Male identity | 0.007 (0.02-0.11) |  |  | 10.3 (0.0-15.3) |  |  |
| Ejaculate identity | 0.005 (0.0-0.12) |  |  | - |  |  |

^a^unpaired males relative to socially-paired males
